# The Impedance Analysis of a Viscoelastic Petalous Structured Stearic Acid Functional Layer Deposited on a QCM

**DOI:** 10.3390/s22197504

**Published:** 2022-10-03

**Authors:** Masruroh, Dionysius J. D. H. Santjojo

**Affiliations:** Department of Physics, Faculty of Mathematics and Natural Sciences, Universitas Brawijaya, Malang 65145, Indonesia

**Keywords:** stearic acid, pillar structures, viscoelastic, QCM, impedance

## Abstract

A functional layer is crucial in a QCM sensor, to immobilize target molecules. The microstructure of the layer determines the sensitivity of the sensor. On the other hand, the microstructure also affects the loading of the sensor. In this study, impedance analysis was used to investigate the relationship between the microstructure and the viscoelastic properties of a petalous stearic acid (SA) functional layer. The SA layer was deposited using a vacuum thermal evaporation technique. Different petalous pillar structures in the elastic layer were generated by varying the deposition time. Analysis showed that the growth of the embedded pillar structures dramatically reduced the conductance and increased the bandwidth. The energy dissipation during the vibration could be related to the interaction between the pillars and the elastic matrix.

## 1. Introduction

Quartz crystal microbalances (QCM) are a type of gravimetric sensor [1]. The advantage of this QCM-based sensor is that the detection of the mass change can reach nanogram scale [2,3,4,5]. Moreover, the QCM has a very fast frequency change response, making it possible to use it in real-time observation. These advantages make QCM sensors very good for gas detection. The sensing working principle is based on the shift of oscillation frequency due to the changes in mass. A QCM is realized by slicing quartz crystals at an AT-cut angle, producing a disk with a shear piezoelectric effect. The crystal oscillates at its resonant frequency (*f_r_*) when supplied with an AC voltage via two electrodes on the disk’s top and bottom. The oscillation is very stable, due to the high value of the Q-factor of the QCM [6]. A functional layer is deposited on the top of the crystal, to entrap the molecules or particles to be measured. The functional layer usually is a rigid thin film, where the relation of the frequency shift (Δ*f*) and the change of mass (Δ*m*) of the sensor can be predicted using the Sauerbrey Equation [7].

(1) $$\frac{\Delta f}{f_{0}}=-\frac{2nf_{0}\Delta m}{Z_{q}}$$

where *n* is an odd-value wave number (*n*= 1,3,5, …) representing the overtone order; and *Z_q_* is the acoustic impedance determined by the active surface area of QCM, the crystal density, and the shear modulus of the crystal [8]. The acoustic impedance for an AT-cut crystal is 8.8 × 10^6^ kg.m^−2^.s^−1^.

Despite its advantages, the drawback of QCM-based sensors is the weak interaction between analyte particles and the sensor surface [9,10,11]. A suitable functional layer on the top of the QCM should be optimized to increase the physical/chemical interaction between the analyte and the sensor [12]. Some functional layers may not have a rigid character in the operational frequency. Instead, they have a viscoelastic behavior, which requires a more complex model. The frequency shift should be modelled as a complex variable (Δf*).

(2) Δf*=Δf+iΔΓ

where Γ is the half-bandwidth at the half-maximum. Using a viscoelastic layer will result in a loss of energy during the oscillations, decreasing the Q-factor or increasing the dissipation factor [13,14].

Currently, the viscoelastic properties of polymeric micro-pillars have been reported by some researchers, who investigated the increased sensitivity of a QCM with a micropillar polymer [2]. The micropillar loading effect and Q factor on the QCM were studied with air and water operation. The cumulative hydrodynamic and micropillar loading was considered, to reduce the Q factor or increase the dissipation energy. Other researchers worked on the effect of the wetting state of liquids on the micropillars [15,16,17,18,19,20]. Their experimental results demonstrated that the wetting state and the micropillar’s height significantly affect the frequency shift of the QCM. Furthermore, the behavior of the micropillars can be used to accurately predict the surface–fluid interaction [21,22].

This work examined the loading mechanism of embedded micropillars of the stearic acid functional layer on QCM sensor performance, by investigating the relation between the morphology of the layer and the related viscoelastic behavior.

## 2. Materials and Methods

### 2.1. Materials and Preparation

The polystyrene layer was made from polystyrene grains obtained from Sigma-Aldrich (Singapore), with a molecular weight of 192 kDa, and dissolved in Toluene solvent to obtain a solution with a concentration of 6%. The polystyrene layer was coated on top of a 10 MHz quartz crystal (QCM) sensor, which functioned as a QCM masking layer, before SA was deposited. The amount of concentration used was calculated using the following formula:

(3) $$\%\;\mathrm{Concentration}=\frac{g\left(\mathrm{solute}\right)}{\mathrm{mL}\left(\mathrm{solvent}\right)}\times 100\%$$

Then the solution was homogenized with an ultrasonic cleaner, coated using a spin coater, and then heated in an oven for 60 min at 200 °C. Furthermore, a layer of stearic acid (SA) was deposited using the thermal evaporation method. SA powder (98% purity) obtained from Sigma-Aldrich was used as the evaporated coating material. The evaporation system was regulated with a voltage of 0.4 V and a current of 17 A. Annealing was carried out for each sample after the deposition process was completed. The annealing was performed at 90 °C in an oven for 60 min. The experiment investigated the microstructure of the layer produced by variation of the effective deposition duration, i.e., 1, 2, 3, and 4 min.

### 2.2. Experimental Method

**Figure 1:**
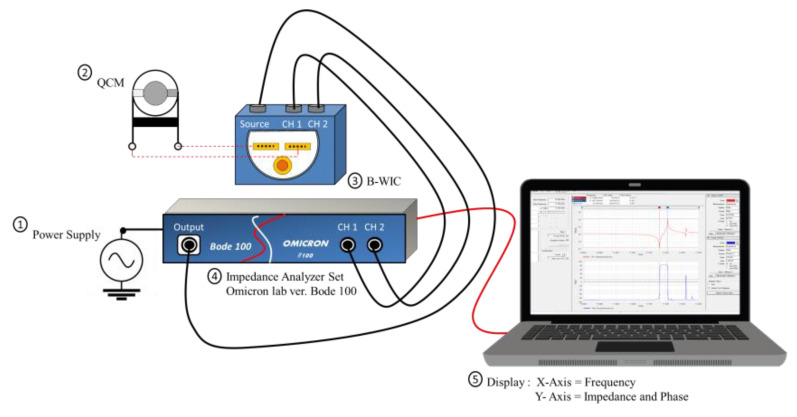
The apparatus for spectroscopic impedance measurements.

An impedance analyzer (Bode 100, OMICRON electronics Asia Ltd., Hongkong) is a device used to measure the complex impedance of a circuit as a function of frequency. The analyzer was connected to a computer as shown in Figure 1. In this study, the impedance measurement results were used to determine the effect of different polymer depositions on the QCM surface on the loading effect. The measurement results were the imaginary and real impedance values of the circuit, frequency, and phase difference.

The morphology of the stearic acid layer was observed using a field emission scanning electron microscope (FESEM Quanta FEG 650, FEI Czech Republic s.r.o., Czech Republic). Observations were carried out from the top and cross-sectional sides.

## 3. Results

**Figure 2:**
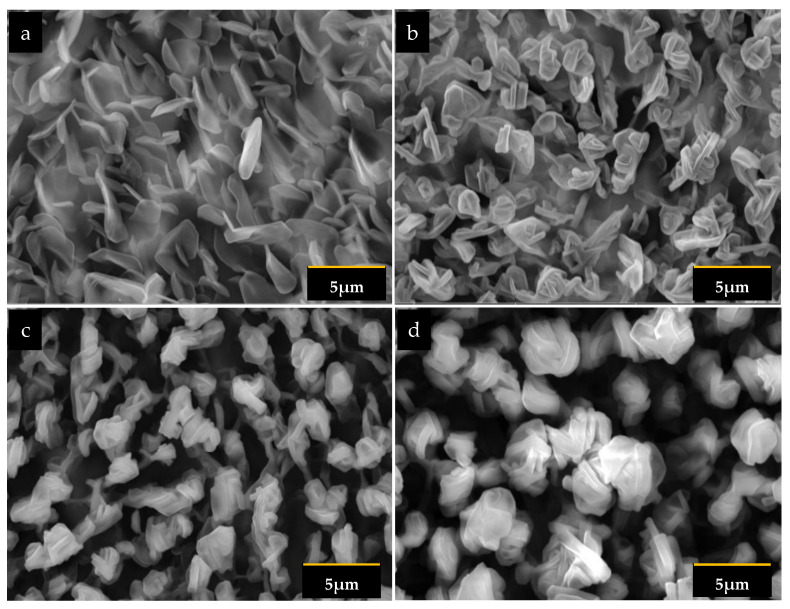
Top view electron micro-image of the stearic acid layer deposited at: (**a**) 1 min; (**b**) 2 min; (**c**) 3 min, and (**d**) 4 min.

The morphology of the stearic acid layer deposited for various times, i.e., one, two, three, and four minutes, is shown in Figure 2.

**Figure 3:**
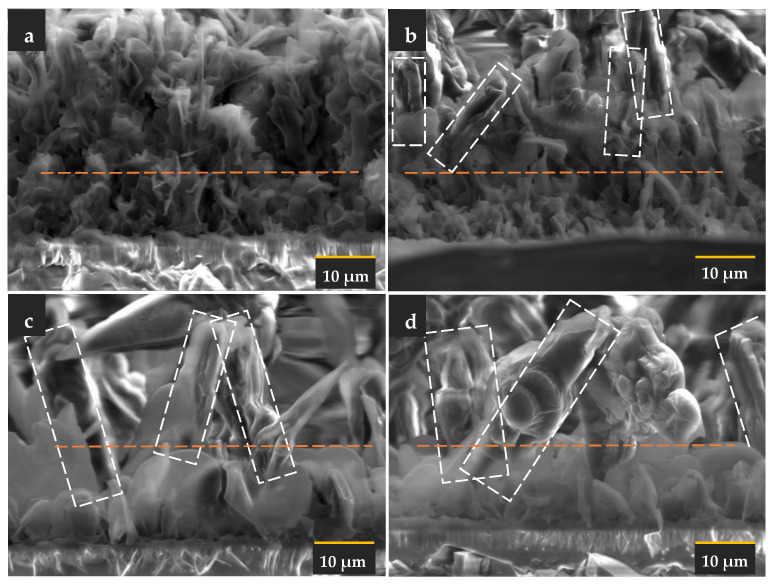
Cross-sectional electron micro-images of the stearic acid layer deposited at (**a**) 1 min, (**b**) 2 min, (**c**) 3 min, and (**d**) 4 min. The white rectangle identifies the pillar-like structure, while the orange line separates the distinguished upper and bottom microstructures.

The layer deposited for one minute had distributed petalous structures. It can be seen from Figure 2a that the petal was relatively large (3.5 nm), and the distribution indicated a relatively higher density structure. A prolonged deposition time significantly affected the morphology of the layer. With a two-minute deposition time, the petal appeared much smaller than at a one minute time (Figure 2b). The large petals disintegrated and agglomerated, resulting in dense, localized clusters. The agglomeration left more empty spaces between the clusters. The agglomerates grew as the deposition time increased, leaving more open spaces and forming a pattern. The petal size was noticeably more extensive at this three-minute deposition time (Figure 2c). Four-minute deposition of the layer increased the petal size, as well as the cluster size. The agglomerate pattern was more perceptible. Further investigations were made by observing cross-sectional micrograph images, as seen in Figure 3.

The cross-sectional observations revealed that the agglomeration pattern may be related to the formation of pillar structures. A pillar was observed in the layer starting at the deposition time of two minutes. It can be inferred that the disintegration and agglomeration process occurred in a vertical direction during the 2 min deposition. The bottom part of the layer did not agglomerate and was distributed homogenously on the substrate. This process was sustained with a longer deposition time. More extensive pillar separation was observed at the three-minute deposition time. The local densification resulted in a less dense overall layer. The micro image of the layer at the four-minute deposition time shows further separation and larger pillars compared to the one with the three-minute deposition time. The image also indicates that the petal at the bottom part of the layer grew into a larger petal as the deposition time increased. However, localization or agglomeration were not observed in the bottom part. It seems that the interfacial layer stayed homogenous during the pillar structure formation.

**Figure 4:**
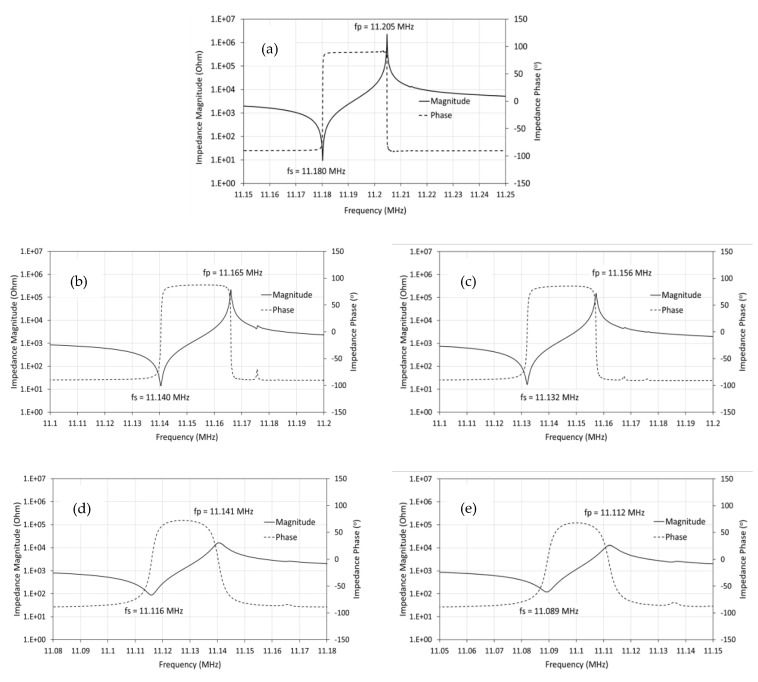
The effect of deposition time on the impedance magnitude and the impedance phase. (**a**) Untreated QCM; QCM coated with SA film deposited for (**b**) 1 min, (**c**) 2 min, (**d**) 3 min and (**e**) 4 min.

The patterned morphology of the layer on the QCM surface can severely affect the sensor’s performance, i.e., the molecular immobilization or trapping and the oscillation damping due to overloading. Generally, the main factors that influence damping are the thickness of the layer and its rigidity or viscoelasticity. The loading behavior of the layer was investigated by measuring the change of the QCM electrical impedance due to the deposition time, as shown in Figure 4. The evolution of the electrical impedance reflects the shift of the layer’s mass distribution and the layer’s mechanical responses.

It can be seen from Figure 4 that the impedance magnitude and phase profile changed dramatically due to the deposition time. Both the series (f_s_) and the parallel (f_p_) resonance frequency were shifted to a lower value, which is common in a loaded QCM. Drastic changes in the profiles can be observed for the three- and four-minute deposited films. Layers deposited for one and two minutes have a similar characteristic profile, which is a firm and pointed peak. On the other hand, those for the three and four minutes are rounded peaks. A pointed peak indicates a tendency for the layer to be rigid, while a rounded one implies a viscoelastic or elastic character; some of the oscillation energy is dissipated by the layer.

## 4. Discussion

**Figure 5:**
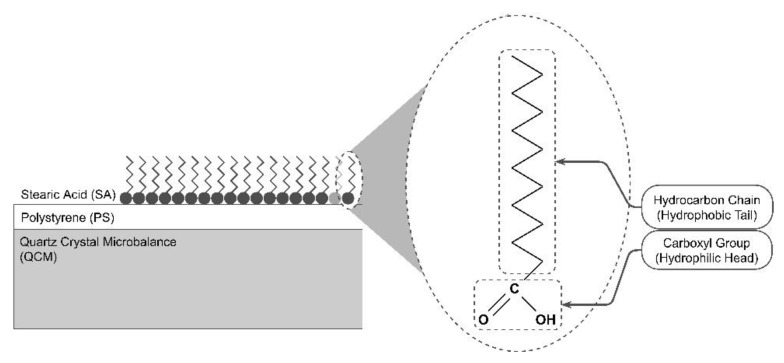
Illustration of the chemical reactions controlling the morphology of the stearic acid layer.

The electron microscope images show that the SA layer’s morphology changed with the deposition time. Initially, the SA layer was dominated by a homogeneously distributed petalous structure, throughout the QCM surface, indicating a strong interaction between the SA layer and the polystyrene masking layer. The SA layer had a compact character and is in the form of a petal spread evenly on the QCM surface. This morphological transformation could occur due to the physical and chemical interactions at the tail and head of the SA molecules, as shown in Figure 5. It is known that the head of a SA molecule is composed of a carboxylic acid formed from a carboxyl group and one remaining group with the formula (R-COOH). The carboxylic acid group can split into an alkoxide (RO-) or carboxylate (RCOO-) anion and a proton radical (H+). These anions are unstable, because there is one unbonded atomic group, so the fraction of this carboxylate group can recombine when interacting with polystyrene molecules ((C_8_H_8_)_n_) to form dihydrogen molecules, through the mechanism of sharing valence electrons. The chemical interaction with shared valence electrons causes the bond between the SA molecules and substrate to become stronger.

**Figure 6:**
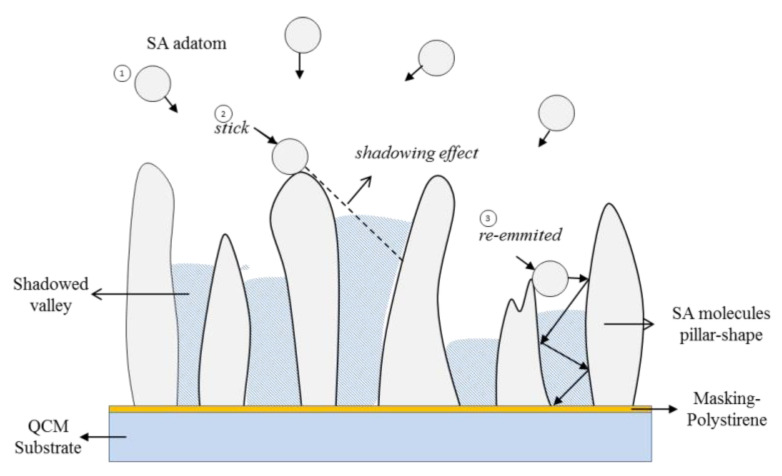
The physical reactions and mechanisms during the deposition control the layer’s morphological change.

In addition, the tail of the SA molecule, composed of carbon chains, plays a role in bringing about physical interactions between SA molecules, in the form of Van der Walls forces. A strong Van der Walls force causes the formation of SA molecular colonies at specific parts of the QCM surface. Then the colonies of SA molecules accumulate to form petalous structures. A longer deposition time of the SA layer (more than 3 min) causes the SA carbon chain to be longer, so that the resulting Van der Walls force will be even greater. The Van der Walls force is enlarged, resulting in the SA molecules increasingly clumping together and accumulating in certain parts. The stack of SA molecules becomes higher and higher, creating a high-low pattern on the QCM surface, such as the pillars shown in Figure 6. The shape of the separated pillars indicates that the SA layer has undergone morphological evolution into the columnar model.

Columnar layer growth is typical in deposition using the PVD method [23,24,25,26,27,28,29]. This mode occurs because the vaporized adatom particles do not necessarily interact with the substrate surface uniformly and evenly. As is known in the gas phase, adatoms will move freely towards the substrate surface with various angles of incidence. When an SA adatom with an angle of incidence that is not perpendicular to the substrate hits the hillside (higher surface) on the substrate, there are two possibilities: that the adatom will stick (stick) or not (non-stick). The adatom attached to the hill area creates a “shadowing effect” during the growth process of the SA layer, causing a part of the substrate to not interact with the adatom. As a result, the longer the deposition is carried out, the taller the hill area, while a steep valley will form in the shadow area. The adatoms not attached to the hill area will be re-emitted from the point of impact to another hill or the shadow valley area. When the SA adatom is reflected in the shadow valley area, over time, the difference in height between the hilltop and valley will be smaller, and the layer will be finer textured.

**Figure 7:**
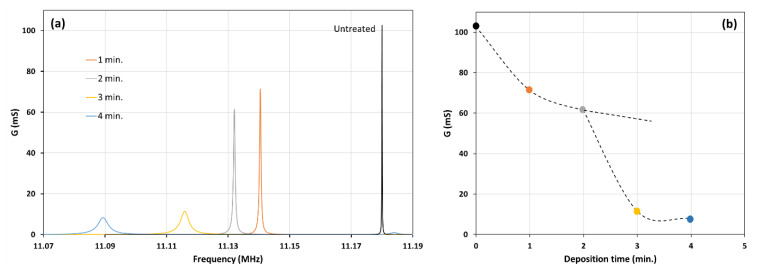
(**a**) The effect of deposition time on the resonance frequency and the conductance. (**b**) The change of the conductance due to the various deposition times.

The impact of the layer’s morphological changes and their characteristics was analyzed using impedance analysis. The impedance measurement of the layers at various deposition times, shown in Figure 4, could be analyzed by calculating the conductance from the data and comparing it for all samples. The results are shown in Figure 7.

As can be seen in the Figure 7b, the conductance dropped drastically for the layer deposited longer than two minutes. This drop as followed by an increased bandwidth and a much larger frequency shift.

**Figure 8:**
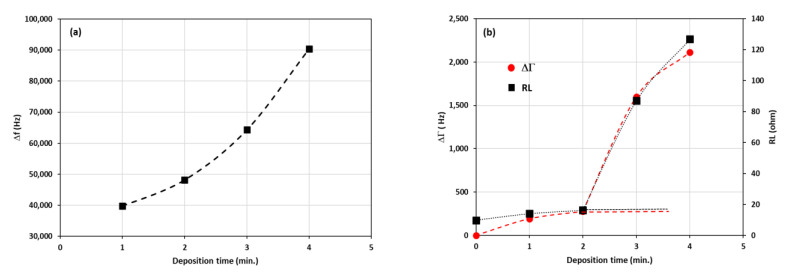
(**a**) The frequency shifts (**Δf**) due to the variation of deposition time. The frequency of the untreated QCM was 11.18 MHz. (**b**) The half-bandwidth changes (**ΔΓ**) and the series load resistance (R_L_) for the layers deposited at the various time. The half-bandwidth of the untreated QCM was 65.9 Hz.

The shift of frequency (**Δf**) was determined by measuring the relative difference compared to the resonance frequency of the untreated quartz crystal (f_0_ = 11.18 MHz). The change in the bandwidth was characterized using the term ΔΓ, as explained earlier, and measured relative to the untreated quartz crystal, i.e., Γ_0_ = 65.9 Hz. The Γ is directly related to the dissipation D = 2Γ/f. The changes can be summarized in Figure 8.

**Figure 9:**
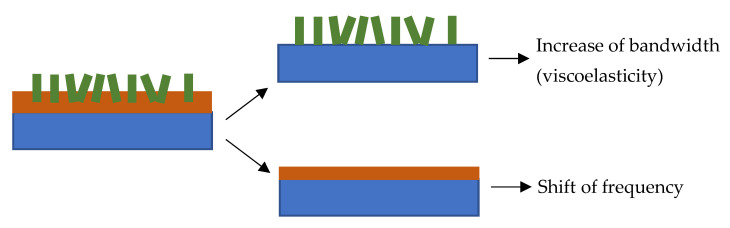
A model describing the morphological composition of the petalous SA layer. The brown layer represents the dense base or matrix component, and the green structure represents the petalous pillar.

It can be seen from Figure 8 that the increase of the frequency shift (Δf) was accompanied by an increase in the change of bandwidth (ΔΓ). Initially, only a slight change was observed for the layers deposited for one and two minutes. The difference was drastically increased for the ones deposited for three and four minutes. The increase in the frequency was related to the deposited mass on the QCM. However, the increase in the bandwidth was not trivial. The bandwidth for a rigid layer was slightly changed with the addition of mass. On the other hand, it changes significantly in a viscoelastic layer [30]. The complex change of the bandwidth in our measurements could be related to the different structures of the layer deposited with various durations. The electron microscope images reveal that the layers consist of two major morphologies, i.e., the dense base or matrix, and the petalous pillars distributed in the matrix. The morphological composition can be described with the model shown in Figure 9.

The model consists of two components, i.e., the base or matrix and the petalous pillars embedded in the matrix. The model on the left side of Figure 9 can be analyzed by separating the composition into two extreme representations on the right side. The first part, the bottom on the right-hand side of the figure, represents the matrix. The second part, the top on the right-hand side of the model, represents the embedded pillars. The matrix alone can be considered a rigid layer that contributes to inertial movements directly related to the system’s resonance. It can be seen from Figure 3 that the inertial layer became denser as the deposition time was increased. Apparently, the inertial layer thickness was also increased significantly for the three-minute and four-minute depositions. This growth directly contributed to the shift of frequency (Δf). At this point, the frequency shift was only considered as the contribution of the mass loading (motional inductance). The contribution of the petalous pillars embedded in the matrix could be related to the change in the bandwidth (ΔΓ).

The composite layer model of the embedded pillar structures could be used to explain the complex viscoelastic character, which was observed from the significant increase in the bandwidth and load resistance. Figure 8b clearly shows the relation between the bandwidth and the load resistance (R_L_) at various deposition times. The increase in load resistance could be connected to the pillar growth as the deposition time was increased. Furthermore, the bonding between the pillars and their surrounding matrix was not rigid. Smaller pillars produced a stronger attachment to the matrix and vice versa. The weaker attachment produced more slippages, releasing energy dissipation (D) during the vibration. As described earlier, the energy dissipation was proportional to the bandwidth (2Γ).

**Figure 10:**
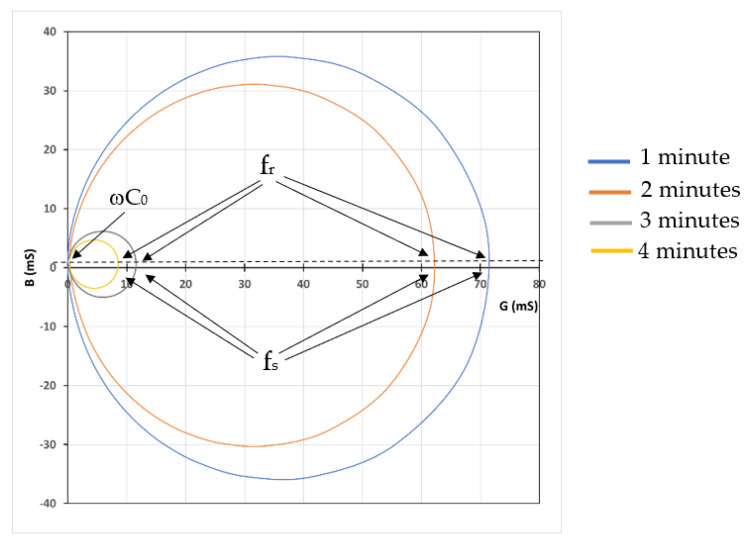
The admittance locus for a QCM with SA layer deposited at various durations. The intersections between the horizontal axis and the circles represent the motional series resonance (f_s_), while the ones between the dotted line and the circles represent the compound resonance (f_r_).

The inclusion of the pillar structures changed the rigid matrix into a viscoelastic layer. The viscoelasticity of the layer was further investigated using an admittance locus diagram, as shown in Figure 10.

It can be seen from Figure 4 and Figure 7 that the compound QCM resonance frequency (f_r_) is close to the motional series resonance frequency (f_s_). The layers deposited at various durations shifted the frequencies (f_r_ and f_s_), but the difference between the two (11 Hz) can be neglected. The fact that f_r_ ≈ f_s_ can be seen in Figure 10. The resonance frequency of the layers (f_r_) was identified at the maximum conductance (G). As can be seen in Figure 10, the maximum conductance occurred at the same susceptance (B). The same susceptance means the layer vibrates in a very similar mode.

Furthermore, the susceptance (B) is closed to zero, indicating that the base layer behaves as a rigid film on the QCM. However, Figure 7 shows that the maximum conductance decreases when the deposition time is increased. The decrease of the conductance, which can also be seen in Figure 10, for the layer deposited in three and four minutes implied a substantial energy loss during the vibration. The locus diagram shows that the positive susceptance areas of the circles are larger than the negative ones. The differences between the positive and negative susceptance areas become more significant for the smaller circles. The negative susceptance represents the storing of energy or the elastic (inertial) loading by the layer, while the positive one represents the storing of energy in the viscous loading. The shift of the circles in the diagram to the positive region indicates more viscous loading due to the petalous pillar structures. Since the structures are embedded in the rigid matrix, the viscoelastic behavior is controlled by the pillar size and their attachment to the matrix, as described previously in Figure 9.

## 5. Conclusions

This work describes the formation of petalous pillar structures during the deposition of a stearic acid layer. The morphology and size of the structures were controlled by the deposition time. Impedance analysis showed that the structures affected the loading resonance frequency and conductance. The embedded pillars in the elastic matrix increased the viscosity of the layer and hence the dissipation. The dissipation was related to the interaction between the pillars and the elastic matrix. A longer deposition time increased the petalous pillars’ size, which may have reduced the attachment. This interaction, however, still needs to be investigated in future work.

## Data Availability

Not applicable.
